# Spatial Distribution of an Uranium-Respiring Betaproteobacterium at the Rifle, CO Field Research Site

**DOI:** 10.1371/journal.pone.0123378

**Published:** 2015-04-13

**Authors:** Nicole M. Koribanics, Steven J. Tuorto, Nora Lopez-Chiaffarelli, Lora R. McGuinness, Max M. Häggblom, Kenneth H. Williams, Philip E. Long, Lee J. Kerkhof

**Affiliations:** 1 Inst. of Marine and Coastal Science, Rutgers University, New Brunswick, New Jersey, United States of America; 2 Dept. of Biochemistry and Microbiology, Rutgers University, New Brunswick, New Jersey, United States of America; 3 Lawrence Berkeley National Laboratory, Berkeley, California, United States of America; University of Coimbra, PORTUGAL

## Abstract

The Department of Energy’s Integrated Field-Scale Subsurface Research Challenge Site (IFRC) at Rifle, Colorado was created to address the gaps in knowledge on the mechanisms and rates of U(VI) bioreduction in alluvial sediments. Previous studies at the Rifle IFRC have linked microbial processes to uranium immobilization during acetate amendment. Several key bacteria believed to be involved in radionuclide containment have been described; however, most of the evidence implicating uranium reduction with specific microbiota has been indirect. Here, we report on the cultivation of a microorganism from the Rifle IFRC that reduces uranium and appears to utilize it as a terminal electron acceptor for respiration with acetate as electron donor. Furthermore, this bacterium constitutes a significant proportion of the subsurface sediment community prior to biostimulation based on TRFLP profiling of 16S rRNA genes. 16S rRNA gene sequence analysis indicates that the microorganism is a betaproteobacterium with a high similarity to *Burkholderia fungorum*. This is, to our knowledge, the first report of a betaproteobacterium capable of uranium respiration. Our results indicate that this microorganism occurs commonly in alluvial sediments located between 3-6 m below ground surface at Rifle and may play a role in the initial reduction of uranium at the site.

## Introduction

**Fig 1:**
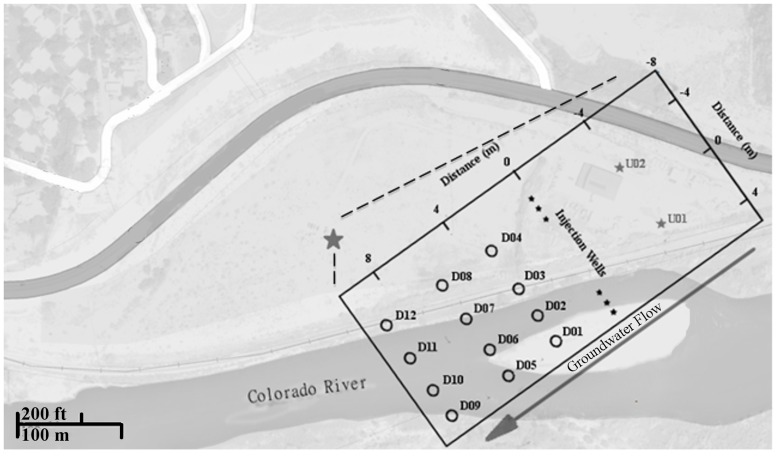
Map of the study area indicating sampling wells and groundwater flow.

The leaching of ore minerals from mining and mill sites has long been a serious problem, resulting in acid mine drainage and polluted run-off to surface and ground waters. One example of ore-processing contamination is subsurface uranium plumes that infiltrate groundwater [1, 2]. Natural attenuation of soluble, oxidized uranium can occur when hexavalent uranium is reduced and precipitated, but the process can be slow due to conditions where suitable endogenous electron donors are limiting. Therefore, recent research efforts have turned to field amendments of electron donors, such as acetate or ethanol, to stimulate microbial uranium (VI) reduction and bring the soluble concentration below safe drinking water standards in a timely manner [3–8]. A principal study site for uranium bioremediation research is the Integrated Field-Scale Subsurface Research Challenge Site (IFRC) in Rifle, Colorado (USA), which is situated near a historic vanadium/uranium mill that operated from the 1920‘s to the 1960’s (Fig 1). Extensive research into the *in situ* bioreduction of uranium has been conducted at the Rifle IFRC [1, 4, 9–14] with field experiments repeatedly demonstrating that groundwater uranium concentrations could be decreased below the U.S. Environmental Protection Agency’s (EPA) drinking water standard of 0.126 μM by adding acetate to the subsurface [1, 3, 15]. The initial decrease in aqueous uranium concentrations occurred concurrently with iron reduction, suggesting that an iron reducing bacterium may be involved in uranium reduction. Substantial shifts in the bacterial community have been documented at the Rifle site following acetate amendment, notably increases in *Geobacter*-like species [4, 9, 11, 12]. In addition, *Geobacter uraniireducens* and *G*. *sulfurreducens* have been isolated from the site or its near vicinity and have been shown to be capable of U(VI) reduction, although these bacteria do not utilize uranium as a terminal electron acceptor [16–17]. In contrast, laboratory experiments (including those using Rifle sediments) demonstrated uranium reduction associated with sulfate-reducing bacteria [18–19]. Likewise, stable isotope probing methods (SIP) have identified a variety of bacteria in the Rifle subsurface which utilize the acetate amended to the groundwater [1, 10, 13], but these active bacteria have not been directly linked to uranium reduction. Unfortunately, none of these studies have conclusively demonstrated growth by a bacterium present at the Rifle site using U(VI) as a terminal electron acceptor or that a particular bacterium capable of uranium reduction is widespread and abundant at the site prior to acetate amendment.

This study was initiated to determine if a microorganism capable of growth on uranium could be isolated from Rifle samples. Using uranyl acetate as an electron acceptor at low concentrations (<10 μM) and acetate as an electron donor, a bacterium, designated strain Rifle, was isolated that could grow on U(VI). The growth of the culture was associated with the reduction of uranium, as evidenced by a partitioning from the soluble into the particulate phase. 16S ribosomal RNA gene analysis indicated the isolated strain was closely related to *Burkholderia fungorum* [20]. The spatial distribution of *Burkholderia fungorum* strain Rifle at the field site was assessed by TRFLP profiling of sediments collected during well installation by employing a diagnostic 4-bp cutter (*MnlI*) and a more definitive 6-bp cutter (*EagI*). The different terminal restriction fragments (TRF) associated with this strain were detected in subsurface sediments at the site prior to acetate amendment. The highest relative contribution to the overall profiles occurred at 3–5 meters depth. Since this microorganism is widely distributed at Rifle and capable of growth using uranium, it may be responsible for a portion of the U(VI) reduction observed during biostimulation with acetate. In addition, understanding the role this microorganism plays in the process of natural immobilization of uranium within the Rifle aquifer can help to elucidate processes inhibiting natural flushing of the aquifer and contributing to the problem of uranium plume persistence at this site and others like it [21, 22].

## Material and Methods

### Field Site

The Rifle IFRC experimental Plot C consists of a gallery of injection (4 cm diameter) /monitoring (10 cm diameter) wells installed in 2007 (Fig 1). Fifty cm thick core sections were collected at 3–6 m below ground surface, bagged, sparged with nitrogen in the field, and transferred to an anaerobic glove bag. Subsamples of sediment (10 g) from the various monitoring wells (D01-D12) were collected in the glove bag and immediately frozen at -80°C in the field prior to transport to the laboratory and processing. A detailed description of the site is found in Williams et al., 2010 [23].

### Isolation of a bacterium capable of growth on uranium

Frozen sediment (1 g) from well D01 was thawed and spiked with 200 μM sodium acetate and 2 μM uranyl(VI) acetate as the sole electron acceptor in a modified minimal carbonate salts media under anaerobic conditions [24, 25]. Modifications included decreasing the NaCl concentration to 2.5 g/L, decreasing the bicarbonate to 1 g/L, and reducing the vitamin and trace salts concentrations by a factor of two. Enrichment cultures were incubated at room temperature for 14 days before transfer. Once slight turbidity was observed in the enrichment, an aliquot was diluted 1:10 with fresh media containing 200 μM sodium acetate and 2 μM uranyl acetate. With each transfer of the enrichment, 1 ml of liquid culture was filtered and extracted for TRFLP analysis [26]. The isolation transfers (7x) were repeated until the enrichment culture consisted of a single 16S rRNA gene TRFLP peak.

### Testing of aerobic growth and identification of the isolated strain

The enrichment was screened for the potential of aerobic growth on Tryptic Soy Agar medium. After streaking onto plates, a large number of colonies with a uniform morphology were obtained. Following 5 rounds of colony purification, an isolate designated strain Rifle was screened by TRFLP again to ensure purity. The 16S rRNA gene from the strain was amplified via PCR using the primers 27F (5'-AGA GTT TGA TCC TGG CTC AG3’) and 1525R (5’-AAG GAG GTG WTC CAR CC-3’), following protocols described previously [27]. The amplicon was sequenced and analyzed via BLAST to detect closely related sequences (99% similarity: 1493/1503 bp identity-to *B*. *fungorum* strain KN-08). The isolate’s 16S rRNA gene (Genbank accession number-KP212894) was used to re-construct a phylogenetic tree with 1366 bp of unambiguously aligned sequence of strain Rifle and 24 closely matching taxa using Geneious software [28].

### Verification of Growth on Uranium as an Electron Acceptor

In order to demonstrate that the isolate was capable of growth on acetate using U(VI) as a terminal electron acceptor, a dosage experiment was established to track changes in cell number associated with increasing concentrations of uranyl acetate. We began by inoculating strain Rifle grown on acetate and uranium (<5 mls) into 1-liter anaerobic minimal media with 200 μM sodium acetate. A schematic of the transfer apparatus that allowed for the anaerobic transfer of aliquots to replicate microcosms is shown in S1 Fig. This design allowed for the continuous sparging of all microcosms during the transfer procedure to minimize oxygen contamination. Initially, subsamples (9 ml) were anaerobically transferred under a headspace of N_2_ /CO_2_ [70:30] to triplicate 10 ml bottles. After these no-uranium amendment control cultures were established, the culture was supplemented with 1μM uranyl acetate and triplicate 9 ml cultures were established for the time course incubations (i.e. 0, 11, and 24 days; n = 9). The culture was then amended with an additional 1 μM uranyl acetate, bringing the concentration to 2 μM and dispensed as above. Further amendments of 3 μM, 5 μM, and 10 μM uranyl acetate were done to complete the experimental treatments. All cultures were incubated under anaerobic conditions (T_0_, T_11_, T_24_ days). Cell numbers were determined by collecting 1 ml of culture from each replicate microcosm at a given uranium amendment and time point (n = 3), preserving with 40 μL of 25% glutaraldehyde, staining with 1% SYBR gold for 15 min, and collected by filtration onto 0.22 μm GE polycarbonate black filters (GE Water & Process Technologies, Trevose, PA USA). Cells were enumerated in 10–15 microscopic fields via fluorescent microscopy using a BH2-RFCA microscope (Olympus, Japan).

### Measurement of uranium by mass spectroscopy

For assessment of uranium concentration in the soluble and particulate fractions within the replicate microcosms, 2 ml of culture at each time point were filtered through 0.025 μm filters (Millipore Corp, Billerica, MA, USA) in an anaerobic chamber (Coy Laboratory Products, Grass Lakes, MI, USA). This soluble fraction (filtrate) was acidified by addition of 0.2 ml nitric acid (70%) and stored in glass vials until analysis. The particulate fraction (filter and the uranium adhering to the walls of the culture vials) were collected by dissolving the filter in 3 ml nitric acid and heating 250°C for 3 hours, or by rinsing the empty culture vials with 3 ml of nitric acid and vortexing for 2 minutes. The uranium concentration was determined by iCAPQ ICP-MS (Thermo Fisher Scientific Inc., Waltham, MA, USA) using Indium as an internal standard. Three iterations per sample were analyzed and RSD percentages averaged 3.6%. A commercial standard (High Purity Standards, Charleston, SC, USA) was used to generate a standard curve for uranium mass based on the instrument signal (counts per second; r^2^ = 0.9955).

### Mapping of the Rifle strain at the study site

Bacterial community composition at the Plot C study site was assessed by 16S rRNA gene TRFLP analysis of DNA extracted from sediment samples collected in 2007. Triplicate nucleic acid extracts were generated from the subsampled sediment (0.25 g) from each core location (n = 12) at 3-6m below ground surface using a high EDTA/phenol/chloroform purification procedure [27, 29]. Amplification of 16S rRNA genes employed the bacterial forward primer 27F (6’-FAM-5'-AGA GTT TGA TCC TGG CTC AG-3’) and a universal reverse primer 1100R (5’-GGG TTG CGC TCG TTG-3’). Cycling parameters were 30 cycles of 94°C for 1 min, 55°C for 0.5 min, and 72°C for 1:10 min, followed by a final extension at 72°C for 10 min. Twenty ng of PCR product were digested for 6 hours with *MnlI* endonuclease and analyzed on an ABI 310 genetic analyzer to visualize the overall bacterial community profile of the sample, and to identify the presence/abundance of the Rifle strain [26]. Verification that samples positive for the strain Rifle *MnlI*-peak (166 bp) represented the *Burkholderia fungorum* strain Rifle isolate was done by digesting a subset of the amplicons using the endonuclease *Eag1* (6-bp cutter) that yields a 215 bp terminal restriction fragment diagnostic of the strain.

## Results

**Fig 2:**
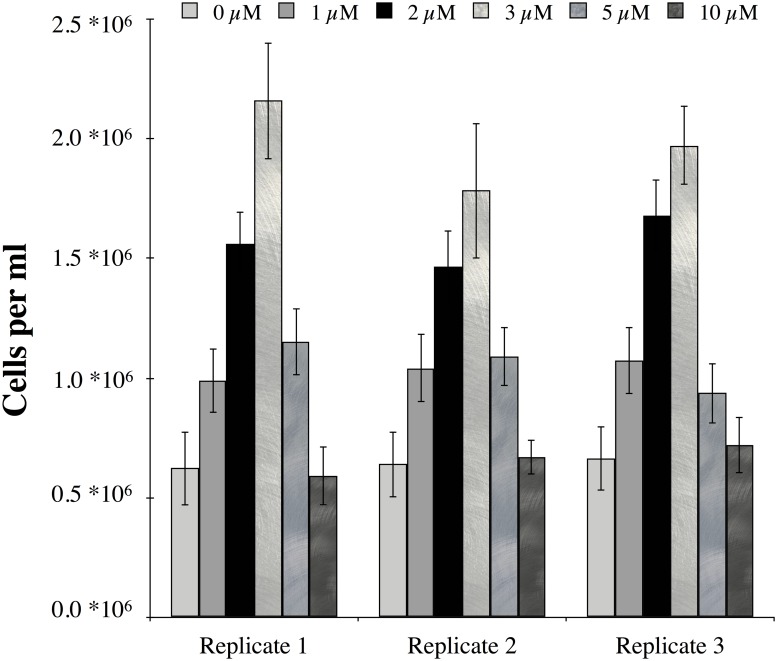
Changes in cell number in replicate microcosms with the various uranyl acetate additions (0–10 μM) after a 24-day incubation. Error bars indicate the variability (SD) in the cell counts for each replicate.

The Rifle IFRC experimental Plot C consists of a gallery of injection (4 cm diameter) /monitoring (10 cm diameter) wells installed in 2007 (Fig 1). Sediment samples from the well gallery were used to establish enrichment cultures on U(VI) minimal media. Colony purification on tryptic soy agar in air yielded a facultative anaerobic isolate with a TRF of 166 bp using *MnlI*. To verify whether this microorganism could grow on U(VI) as a terminal electron acceptor under anaerobic conditions, a dose-dependent experiment was established using increasing uranyl acetate concentrations (1–10 μM) with a uniform amount of acetate as electron donor (S1 Fig). The initial inoculum contained 0.45 ± 0.08 x 10^6^ cells/ml. After 24 days in the uranium amendments, the control (0 μM, i.e. no uranium amendment) displayed a slight increase in cell numbers to 0.6 ±. 14 x 10^6^ cells/ml (Fig 2). Each 1 μM uranium increment up to 3 μM yielded an increase of 0.5 ± .13 x 10^6^ cells/ml. For the 5 and 10 μM amendments, lower cell numbers were observed, suggesting toxicity at the higher uranium dose.

**Fig 3:**
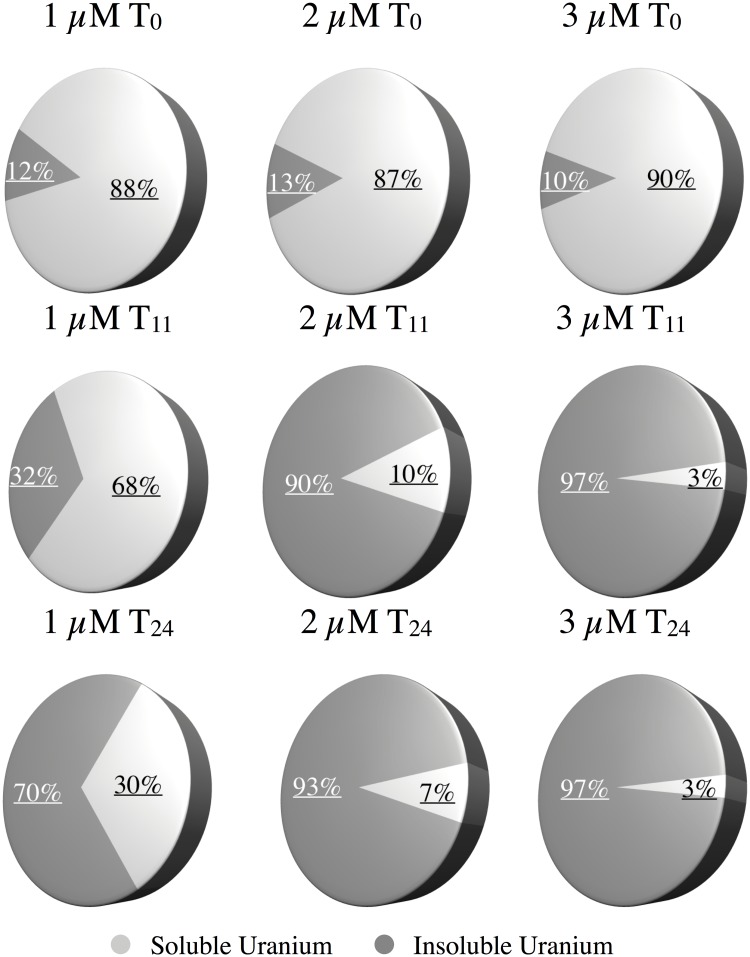
Average proportion of soluble and insoluble uranium over time with uranyl acetate addition (0–3 μM) measured by ICP-mass spectrometry.

Verification that the increase in cell number of the pure culture was linked to the reduction of uranium was done by quantifying the fraction of uranium in both the soluble and insoluble pools. Uranium in the cultures spiked to 3 μM shifted from ~90% soluble at the T_0_ time point for all 3 concentrations to 70–97% insoluble by the end of the 24 day incubations (Fig 3). A mass balance indicated that 93–102% of the added uranium could be accounted for in the soluble/insoluble pools. Interestingly, the bacterium also reduced uranium as efficiently at 5 and 10 μM concentration (up to 97%-S2 Fig). However, the percent of soluble uranium at the T_0_ sampling time point was 78% for the 5 μM and 18% for the 10 μM treatments. Presumably, the bacteria were stimulated by the prior exposure to uranium during transfer and began reducing the radionuclide before the T_0_ samples could be collected.

To ensure that the U(VI) was reduced (rather than adhering to cells or abiotically precipitating with phosphates in the media) a separate growth experiment with 2 μM uranyl acetate was conducted as described above. However, subsamples from this time course were filtered through 0.025 μm filters in air and not in the anaerobic chamber. The results demonstrate the uranium is largely in the soluble fraction (>90%) when exposed to oxygen (S3 Fig) and verify that the U(VI) is reduced to U(IV) during growth by strain Rifle.

**Fig 4:**
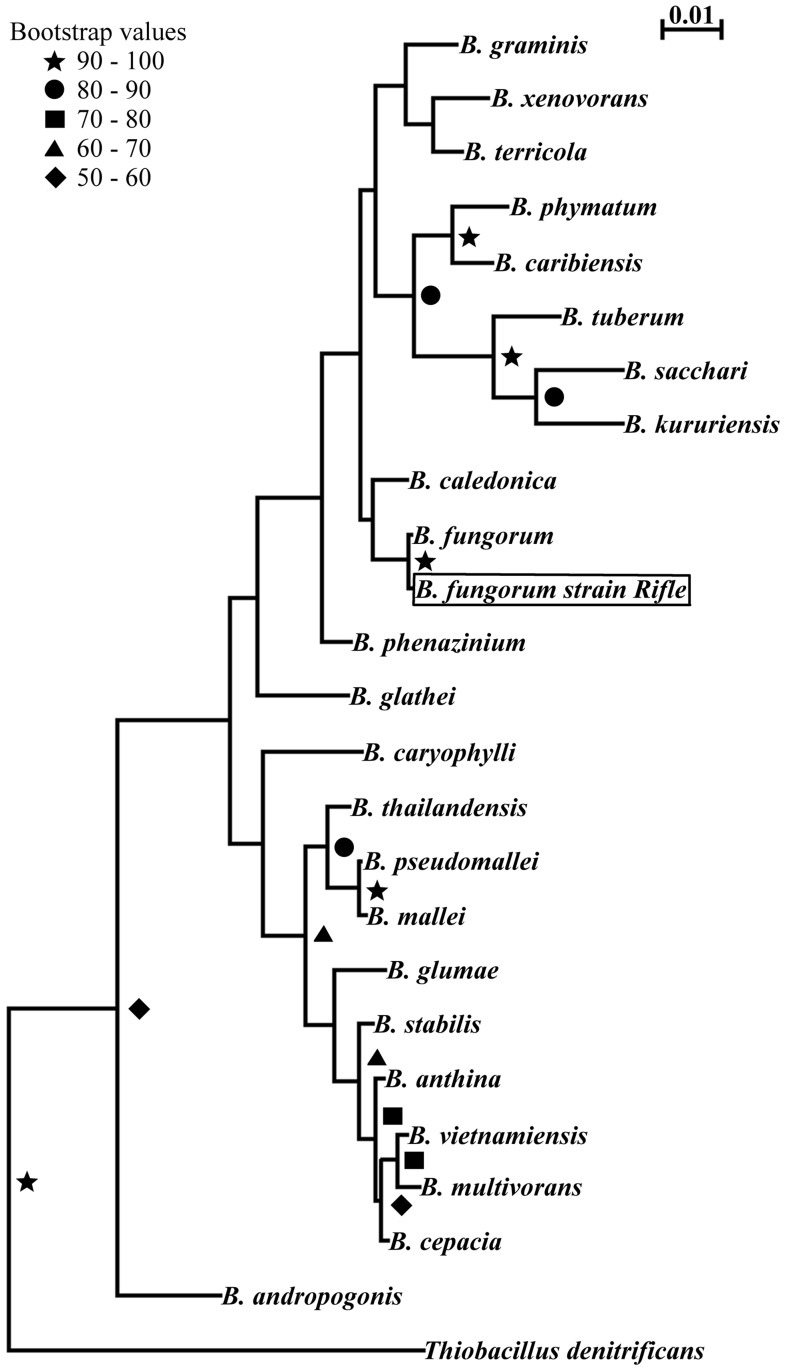
Maximum likelihood phylogenetic tree re-construction of *Burkholderia* type strains using 1366 bp of unambiguously aligned sequence of the 16S rRNA gene. *Burkholderia fungorum* strain Rifle is indicated.

**Fig 5:**
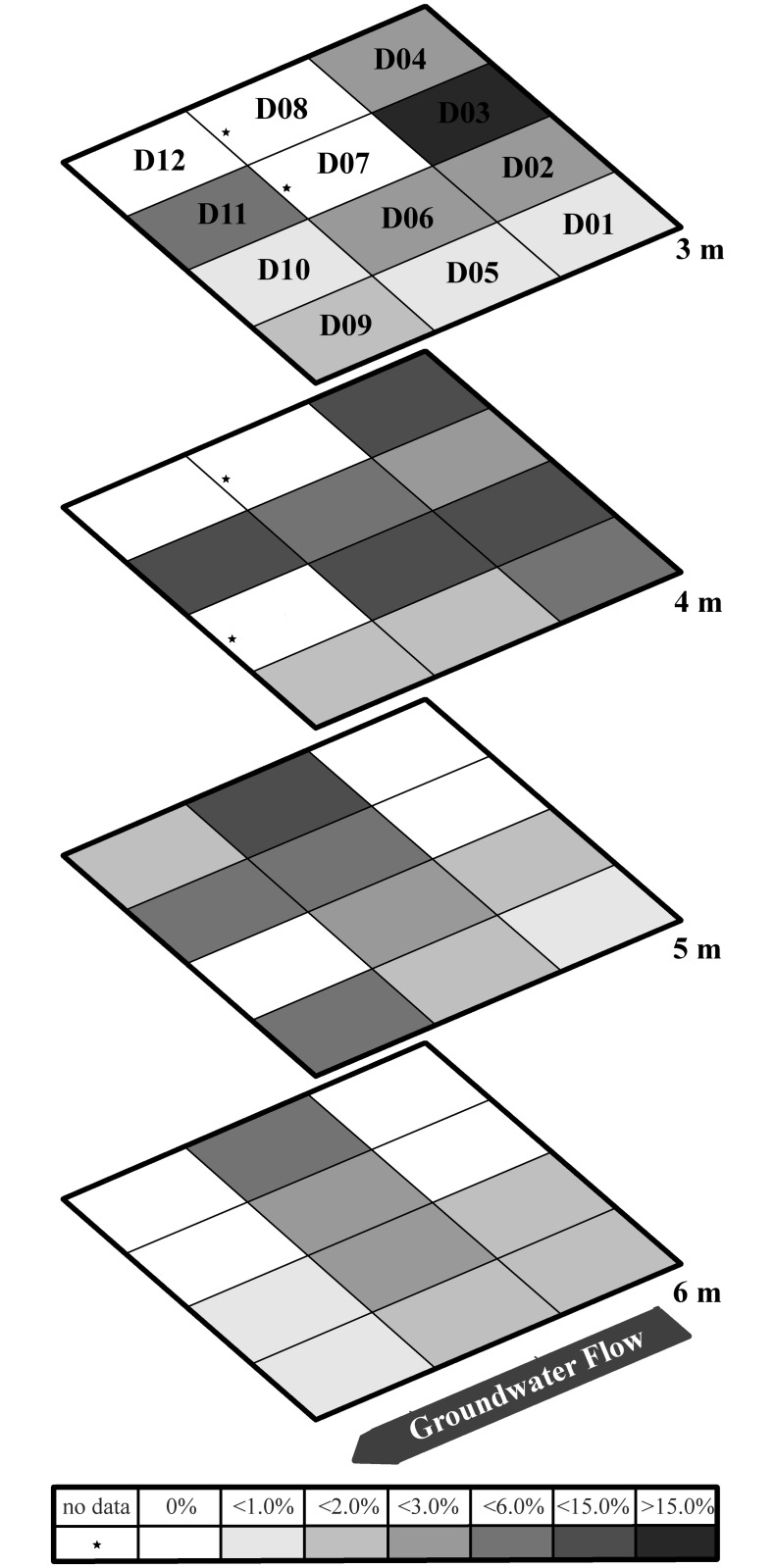
Spatial distribution of *Burkholderia fungorum* strain Rifle based on percent contribution to overall microbial community using the 166 bp MnlI peak in the bacterial TRFLP profile.

To determine the identity of strain Rifle, 16S rRNA sequence analysis was performed. BLAST results indicated the Rifle isolate was closely related to members of the *Burkholderia* genus. A phylogenetic tree of strain Rifle and other bacterial taxa places the Rifle strain within the *Burkholderia* cluster, closest to *B*. *fungorum* (Fig 4). The spatial distribution of *Burkholderia fungorum* strain Rifle within the field gallery was determined by TRFLP analysis of 16S rRNA genes in sediments recovered during well installation. These sediments were not field amended with acetate and represent the microbial community prior to perturbation to stimulate uranium reduction through organic carbon injection. A 166 bp TRFLP peak in 16S rRNA gene profiles from the site using *MnlI* was considered diagnostic of *Burkholderia fungorum* strain Rifle in the various samples. The contribution of OTU 166 to the overall bacterial community is presented in Fig 5. The relative abundance of *Burkholderia fungorum* strain Rifle ranged from 0–15% in the community profiles within these sediments. The 166 peak was most abundant in the upper part of the soil column (3 and 4 m depth) and the signal generally diminished at lower depths (6 m). The highest contribution of the 166 bp peak to the overall community (>15%) was in well D03 at 3 m. The patchy nature of the *Burkholderia fungorum* strain Rifle distribution can be seen in many wells where the relative contribution to the microbial community could vary from <15% to <1% within 1 to 2 m. To test whether the 166 bp peak might represent more than one 16S rRNA gene, a second set of enzyme digests of the fluorescent amplicons was performed using a 6-bp cutter (*EagI*), yielding a 215bp TFLP peak for *B*. *fungorum* strain Rifle. This analysis produced the appropriate sized TRF for all samples analyzed and the *EagI* 215 bp peak area was 1–50% of the original *MnlI* 166 peak area.

## Discussion

The reduction of soluble U(VI) to less soluble U(IV) species is an essential step in the redox immobilization of this toxic radionuclide in contaminated groundwater. Initially, uranium reduction was believed to be entirely abiotic in nature, with organic and inorganic compounds acting as reducing agents. This notion changed in the early 1990’s when biotic uranium reduction was described for a dissimilatory Fe(III)-reducing bacterium [30]. Since then, numerous bacteria have been found which are capable of reducing uranium [31]. Of these bacteria, only *Anaeromyxobacter dehalogenans; Carboxydothermus ferrireducens*, *Desulfotomaculum reducens*, *Geobacter metallireducens*, and *Shewanella putrefaciens* have been reported to grow using U(VI) as a terminal electron acceptor [30–35]. More often, a resting cell assay at 200–10,000 μM is used to assess uranium reducing potential and over 30 bacteria have been described that reduce U(VI) to U(IV) but are not necessarily capable of growth on uranium [31, 36]. In addition, mixed cultures have demonstrated uranium reduction under co-metabolic conditions with terminal electron acceptors including sulfate [37] or iron [16, 30, 38–40].

Because so few of the known bacteria capable of growth on uranium have been detected at the Rifle IFRC site, much of the effort has focused on the role of *Geobacter*-like organisms during the course of acetate amendment and bioremediation. Yet, stable isotope probing experiments indicated that a variety of bacterial species are stimulated by the amendment of acetate at Rifle [1, 10, 13]. Interestingly, one of the major bacteria discovered at Rifle that incorporates ^13^C-acetate also displayed a 166 bp peak (using *Mnl I*) in the active community profiles [13]. This earlier report identified the 166 bp peak as an alphaproteobacterium from clone libraries. Here, we suggest that the 166 bp peak in the unamended sediment could also be *Burkholderia fungorum* strain Rifle. This result is consistent with our digestion of the TRFLP amplicons using *EagI*, suggesting that 1–50% of the *Mnl I* 166 bp peak area as some other microorganisms (such as the previously described alphaproteobacterium).

Furthermore, a next generation 16S rRNA gene analysis of samples taken from the Rifle site indicated that *Burkholderiales* comprised nearly 30% of the subsurface microbial community prior to field amendment [41]. *Burkholderia-*like microorganisms have also been shown to be a substantial proportion of the microbial community in uranium reducing enrichments from the Oak Ridge, TN IFRC site [6, 42, 43]. As with Rifle, the beta-proteobacteria detected after enrichment at Oak Ridge were not thought to play a role in uranium reduction. However, the fact that *Burkholderia fungorum*. strain Rifle grows on U(VI) is not entirely surprising as this genus is well documented for its wide variety of metabolic capabilities. For example, *Burkholderia* species have been found to be capable of anaerobic growth on nitrate, including *B*. *fungorum* [44, 45], *B*. *xenovorans* [46] and *B*. *pseudomallei* [47]. In addition, many members of the genus have been isolated based on the ability to degrade a broad range of carbon compounds [48–54]. This metabolic flexibility by *Burkholderia* species has been attributed to the large differences in their multiple genomes, varying as much as 2 Mbp between strains [55, 56]. Interestingly, a preliminary study of the terminal electron acceptor preferences by *B*. *fungorum* strain Rifle indicated only oxygen and uranium are respired on M9 media with dextrose as an electron donor (S4 Fig). This study suggested *B*. *fungorum* strain Rifle does not grow on iron, in contrast to all other known bacteria capable of growth on uranium which are also capable of utilizing iron as a terminal electron acceptor. These results imply a genomic comparison of our isolate with other related *Burkholderia* species that cannot grow on uranium should provide additional insight into the genes and mechanisms of biotic uranium reduction in this genus and may represent a novel respiratory pathway for radionuclide reduction.

In conclusion, *Burkholderia fungorum* strain Rifle is the first microorganism isolated specifically from the Rifle IFRC site that is capable of growth on U(VI), and exhibits an identical growth yield to uranium as *Anaeromyxobacter dehalogenans* [35]. Previously, members of the *Burkholderiaceae* were not suspected to play a role in uranium reduction and immobilization. Given the long period of uranium contamination at the site (>60-years), even slow rates of natural uranium reduction by *Burkholderia fungorum* strain Rifle and similar microorganisms could be expected to immobilize a potentially significant pool of uranium within the aquifer and can lead to uranium plume persistence. Such immobilization has the potential to impede the groundwater compliance strategy for Rifle and similar mill tailings impacted sites, which rely on natural flushing of the aquifer by low uranium groundwater to remove residual contamination. This study suggests that the ability to respire uranium could be widespread among facultative anaerobes and testing to date is often performed under conditions that could be toxic to many microorganisms. Furthermore, our findings highlight how integrated research at DOE field sites can lead to the discovery of novel metabolic capabilities in different microorganisms and to new ideas for promoting biostimulation and uranium reduction at these contaminated sites.

## Supporting Information

S1 Fig Experimental apparatus for distributing a uniform inoculum for the various uranium additions while maintaining anaerobic conditions. (TIFF)

S2 Fig Average proportion of soluble and insoluble uranium over time with uranyl acetate addition (5 and 10 μM) measured by ICP-mass spectrometry. (TIFF)

S3 Fig Uranium solubility when anaerobic culture samples are filtered under oxidizing conditions. (TIFF)

S4 Fig Changes in cell number in replicate microcosms with various terminal electron acceptors after a 5-day incubation. Cells were grown in M9 media with 0.4% dextrose as an electron donor and the terminal electron acceptor indicated. Error bars indicate the variability (SD) in the cell counts for each microcosm. The inset is the anaerobic incubations on a different scale.
(TIFF)
