# Supplementary figures and images for: Spatial Distribution of an Uranium-Respiring Betaproteobacterium at the Rifle, CO Field Research Site
Source: PLoS One. 2015 Apr 13;10(4):e0123378. doi: 10.1371/journal.pone.0123378 (PMC4395306; doi:10.1371/journal.pone.0123378)

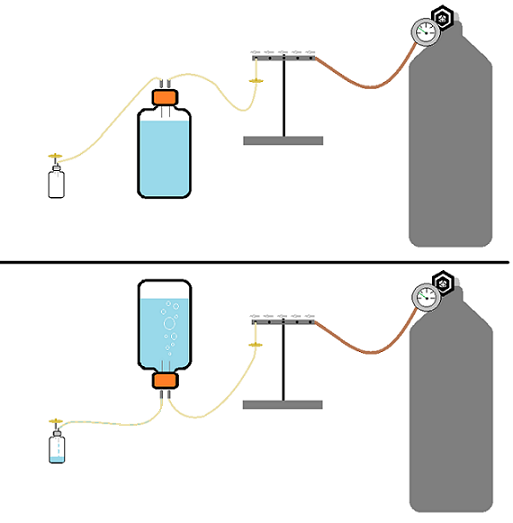

Supplement: S1 Fig — (TIFF) [file pone.0123378.s001.tiff]

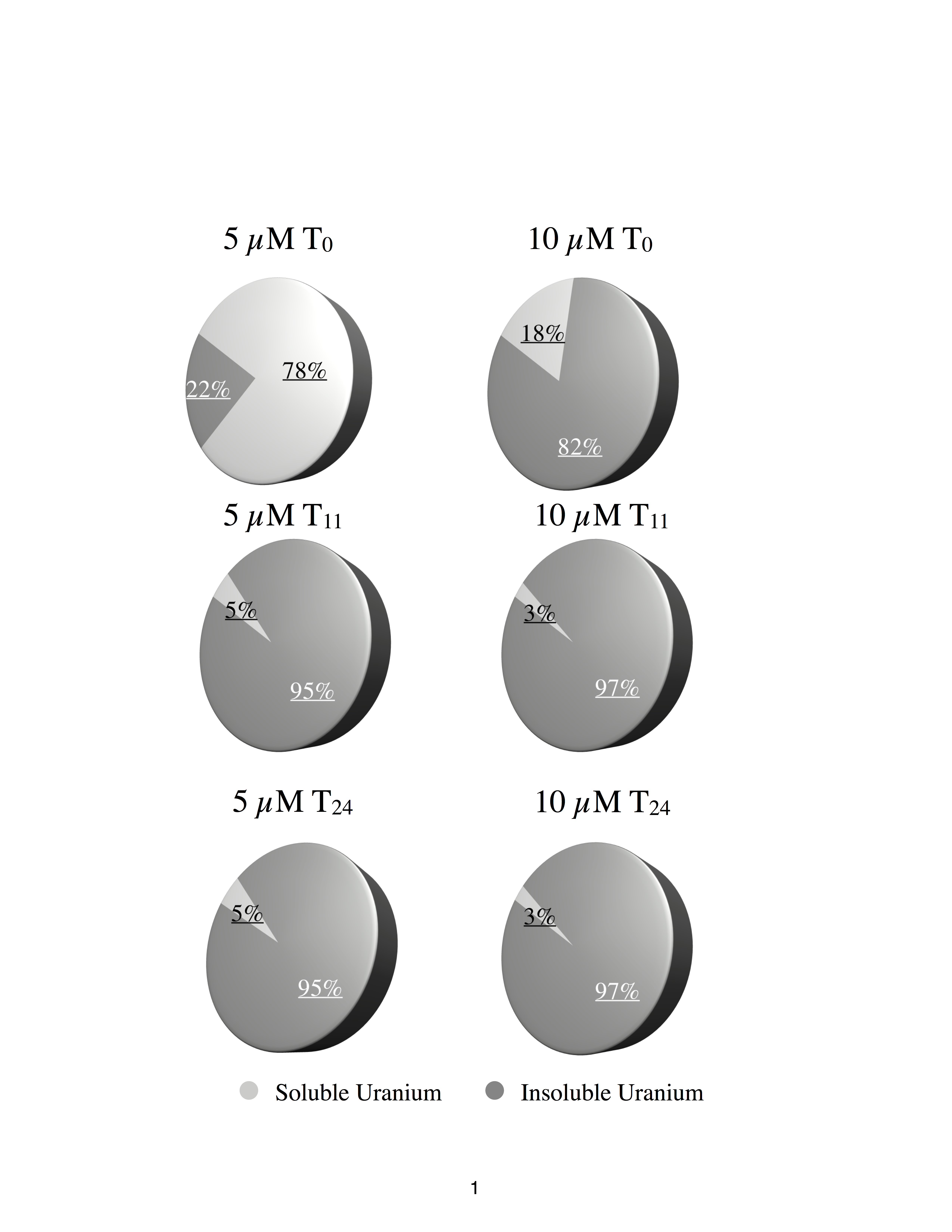

Supplement: S2 Fig — (TIFF) [file pone.0123378.s002.tiff]

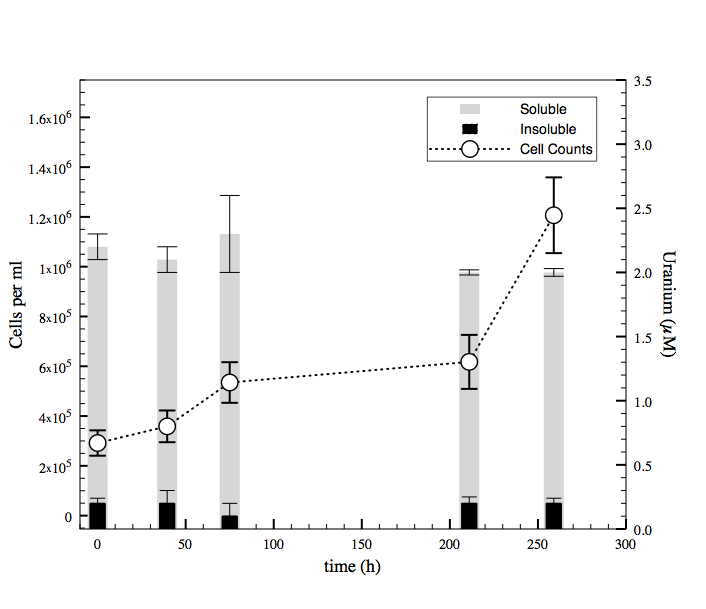

Supplement: S3 Fig — (TIFF) [file pone.0123378.s003.tiff]

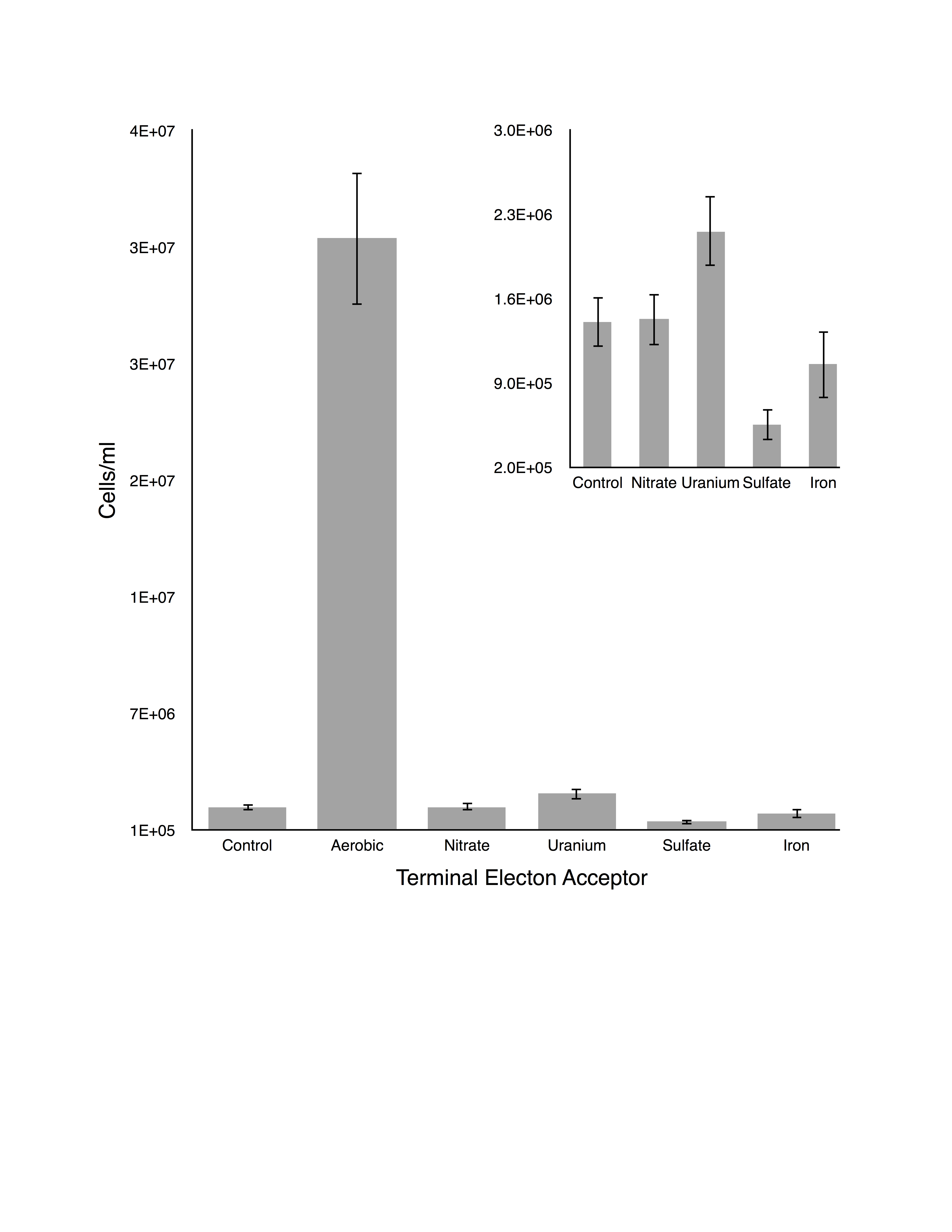

Supplement: S4 Fig — Cells were grown in M9 media with 0.4% dextrose as an electron donor and the terminal electron acceptor indicated. Error bars indicate the variability (SD) in the cell counts for each microcosm. The inset is the anaerobic incubations on a different scale. (TIFF) [file pone.0123378.s004.tiff]
